# Landscape review of current HIV ‘kick and kill’ cure research - some kicking, not enough killing

**DOI:** 10.1186/s12879-017-2683-3

**Published:** 2017-08-29

**Authors:** Kristian Thorlund, Marc S. Horwitz, Brian T. Fife, Richard Lester, D. William Cameron

**Affiliations:** 1 Department of Health Research Methods, Evidence and Impact, Faculty of Health Sciences, McMaster University, Ontario, Canada; 2 Faculty of Microbiology and Immunology, University of British Columbia, Vancouver, Canada; 3 Department of Medicine, Center for Immunology, University of Minnesota, Minneapolis, Minnesota 55455 USA; 4 Department of Medicine, University of British Columbia, Vancouver, Canada; 5 Faculty of Medicine, University of Ottawa, Ottawa, Ontario Canada; 6 Division of Infectious Diseases, Department of Medicine, University of Ottawa at The Ottawa Hospital / Research Institute, 501 Smyth Road, Ottawa, K1H 6V2 Ontario Canada

## Abstract

**Background:**

Current antiretroviral therapy (ART) used to treat human immunodeficiency virus (HIV) patients is life-long because it only suppresses de novo infections. Recent efforts to eliminate HIV have tested the ability of a number of agents to reactivate (‘Kick’) the well-known latent reservoir. This approach is rooted in the assumption that once these cells are reactivated the host’s immune system itself will eliminate (‘Kill’) the virus. While many agents have been shown to reactivate large quantities of the latent reservoir, the impact on the size of the latent reservoir has been negligible. This suggests that the immune system is not sufficient to eliminate reactivated reservoirs. Thus, there is a need for more emphasis on ‘kill’ strategies in HIV cure research, and how these might work in combination with current or future kick strategies.

**Methods:**

We conducted a landscape review of HIV ‘cure’ clinical trials using ‘kick and kill’ approaches. We identified and reviewed current available clinical trial results in human participants as well as ongoing and planned clinical trials. We dichotomized trials by whether they did not include or include a ‘kill’ agent. We extracted potential reasons why the ‘kill’ is missing from current ‘kick and kill’ strategies. We subsequently summarized and reviewed current ‘kill’ strategies have entered the phase of clinical trial testing in human participants and highlighted those with the greatest promise.

**Results:**

The identified ‘kick’ trials only showed promise on surrogate measures activating latent T-cells, but did not show any positive effects on clinical ‘cure’ measures. Of the ‘kill’ agents currently being tested in clinical trials, early results have shown small but meaningful proportions of participants remaining off ART for several months with broadly neutralizing antibodies, as well as agents for regulating immune cell responses. A similar result was also recently observed in a trial combining a conventional ‘kick’ with a vaccine immune booster (‘kill’).

**Conclusion:**

While an understanding of the efficacy of each individual component is crucial, no single ‘kick’ or ‘kill’ agent is likely to be a fully effective cure. Rather, the solution is likely found in a combination of multiple ‘kick and kill’ interventions.

## Introduction

Even though human Immunodeficiency virus (HIV) was identified as the cause of Acquired Immunodeficiency Syndrome (AIDS) over 30 years ago, we still do not have a general cure [1]. Of the estimated 71 million people infected to date, only one documented patient, the Berlin Patient, is believed to have been cured [2]. In this case, the cure was achieved by exploiting the radical measures required to treat the patient’s acute myeloid leukemia. While inspiring to cure enthusiasts, this approach is, however, not applicable to the broader population. Nevertheless, the case of the Berlin patient did propel new interest in curative HIV research approaches.

Most cure research efforts to date have been rooted in the so called “Kick and Kill” approach – an approach that is based on the premise the HIV virus partially ‘hides out’ in so-called latent reservoirs and that activating these latent reservoirs will result in the destruction of the reactivated cells either by attack by the immune system, or by the cytotoxic effects of HIV itself. To date, however, clinical trials employing kick and kill approaches have yet to deliver promising results.

In this article, we review what is currently known about viral transmission under antiretroviral therapy (ART) and the mechanisms underlying kick and kill approaches. We conclude that kick and kill has mainly focused on the ‘kick’ component and neglected the ‘kill’ component. We then review available strategies for the ‘kill’ component and summarize a potential approach to complete the kick and kill for effective therapy.

### What is currently known about viral transmission, viral memory and ART?

Today, HIV/AIDS is a manageable, livable disease with many antiretroviral drugs available that safely and effectively suppress plasma viremia and maintain adequate peripheral blood CD4^+^ T-lymphocyte counts. However, effective treatment does not clear the virus infection, and its suppression requires lifelong treatment. This is because the current drugs impair the various stages of the viral lifecycle (viral entry to a target cell, reverse transcription, DNA integration, protein cleavage), but do not affect infected cells when these processes are not active. In general, when the immune system gains control over an infection, which is signalled by antigenic clearance, active inflammation and immunity diminish and a memory of specific immunity comprised of residual long-lived ‘antigenically committed’ memory T-cell remains. This memory can rapidly mount an anamnestic T-cell response upon re-exposure to familiar antigens. For HIV, the immune system never gains suppressive control of the infection or clearance of the virus. Rather, in the attempt to generate HIV-specific immunity, several of memory T-cells generated to ensure effective future immune responses remain infected because the active CD4+ T-cell from which they differentiated were already infected. Thus, a long-lived reservoir of HIV-infected memory T-cells is established. Most of these cells are not affected by current anti-retroviral therapies, and can hang around in significant numbers in an inactive, or quiescent state for decades with an estimated half-life of about 3½ years on ART [3, 4]. It has been estimated that systemically, there are somewhere in the range of 10^6^–10^7^ replication competent infected latent CD4+ T-lymphocytes, capable of rapidly re-establishing the viral population upon withdrawal of ART [5]. In addition, recent evidence that a significant proportion of clonally expanded memory T-cells are replication competent [4, 6, 7]. Memory T-cells are therefore considered to be the most important source of persistent infection.

From the perspective of HIV cure research, it is essential to keep in mind that other cell types can become infected by HIV. Particularly, monocytes and macrophages are thought to be of importance, as they have been shown to harbour large quantities of viral particles within intracellular endosomes [8–11]. These cells are long-lived (weeks to years), can infiltrate multiple tissue compartments including the central nervous system, are resistant to the cytolytic effects of HIV infection, can produce significant amounts of virus, and are believed be a key contributor of cell-to-cell transmission, even during ART [12–15]. In addition, the direct effects of ART treatment on macrophages is complex and not fully understood [16]. For example, a recent (April 2017) ex vivo study on 9 myeloid-only mice infected with M-tropic HIV and treated with 7 weeks of ART showed conventional viral rebound in 3 mice (33%) after interruption of ART [17]. However, this study does not explain the reason for viral rebound in 33% of the mice. Macrophage can be found in every tissue in the body as specialized subsets, and are not likely to receive uniform exposure to the drugs systemically. In the aforementioned study, there was a weak trend of a comparably lower number of macrophages in the spleen tissue and higher number of macrophages in the bone marrow among the 3 mice with viral rebound. However, larger sample sizes are required to validate this observation. Lastly, nucleoside reverse transcriptase inhibitors (NRTIs), non-nucleoside reverse transcriptase inhibitors (NNRTIs) and protease inhibitors (PIs) are less potent in these cells during chronic infection as determined by the half maximal effective concentration (EC_50_) and might be expected to be less suppressive.

Follicular dendritic cells, which are critical cells found in the B-cell follicles of the lymph nodes and other lymphoid tissues, are also considered to be an important viral reservoir. This is predominantly because they can shelter viral particles within endosomal compartments or carry multiple particles attached out outer membrane synapses. These particles can then be delivered to large populations of uninfected CD4+ T cells inside the lymph nodes, thus causing their infection and viral spread [18, 19]. While ART does reduce the viral titer within this reservoir, evidence suggests that viral clearance is not complete [20]. The contribution of these reservoirs to plasma viremia during natural infection or viral rebound when ART is interrupted is not known. Thus, we propose a focused evaluation of reservoirs would be important when studying cure approaches. At this point, we should acknowledge that research on viral reservoirs, whether latent memory T-cells, macrophages, or dendritic cells, presents several challenges due to the limited sensitivity of the assays and techniques currently available to quantify them. We therefore recommend this article be read in the light of this general limitation of HIV ‘cure’ research.

In the case of the Berlin patient, treatment for acute myeloid leukemia took place over several months and included lymphomyeloid ablative chemo- and radiation therapies, which are believed to have purged the patient’s T-cells [2]. As a result, two stem cell transplants were administered from a tissue compatible allogenic donor (to limit graft-versus-host disease) for immune system reconstitution. The donor of the bone marrow was electively selected to carry a homozygous delta32 deletion in CCR5 gene. During the treatment, a grade I level graft-versus-host disease was observed localized to the skin. Minor adjustments in cyclosporine modified and improved the disease and it is unclear if the graft-versus-host episode was a co-factor in the elimination of virus. While the precise mechanism by which the Berlin patient was cured is not clear, it is generally assumed that the above treatment led to the elimination of the vast majority (if not all) of this latent reservoir, as 10 years (February 2017) has not lapsed since ART was interrupted.

### Kick and kill strategies to date

While there are many exciting cure strategies being pursued, the so called “Kick and Kill” approach has received the most attention. This approach is based on the premise that activating the latent reservoir will result in the destruction of the reactivated cells either by attack by the immune system, or by the activation-induced cell death associated with viral production in active CD4+ T-cells. At the same time, expansion of the infection is expected to be suppressed by concomitant administration of ART. Thus, it was believed that simply ‘kicking’ latent cells into activity might be enough to lead to the elimination of the latent reservoir. This is not a new idea. In fact, the first attempts at activating the reservoir were carried out over a decade ago and involved treatment with recombinant cytokines. For example, Interleukin 2 (IL-2) has been the subject of a number of studies in humans because of its ability to effect T-cell activation, proliferation and survival; however, the observed clinical effect at tolerable doses did not warrant further pursuit of these relatively toxic therapies [21, 22]. Since the first studies on cytokines, several sophisticated T-cell reservoir activation strategies leveraging our increasing knowledge of the mechanisms that preserve latency have been attempted. Two such approaches include: 1) treatments targeting the release of chemically sequestered cellular transcription factors essential to the initiation or propagation of viral transcription (e.g. NF-αB, NFAT, P-TEF, AP-1) and 2) epigenetic modulation of the HIV promotor site to favour HIV transcription. Below, the evidence on individual agents that fall within these two strategy categories are outlined. Further Table 1 presents and overview of ‘kick’ strategies that have been tested in clinical trials in humans to date.

**Table 1:** Overview of ‘kick’ studies in humans completed to date

Study	Drug Studied	Patient Eligibility	No. Pts	Study Design	Tx Dose and Duration	Length of Study	Key findings
Agents targeting cellular transcription factors							
Lehrmann 2005	Valproic Acid (HDACi)	Viral RNA < 50cp/mL for at least 2 years	4	Proof-of-concept	500-750 mg bid for 3 months	18 weeks	68%–83% reduction in resting infected CD4 T-cells in 3 of 4 patients
Sagot-Lerolle et al. 2008	Valproic Acid (HDACi)	Viral RNA < 50cp/mL for at least 2 year	24	Retrospective matched cohort study (pilot)	Not reported (retrospective)	2 years	No difference in viral DNA quantified in PBMCs
Archin 2010	Valproic Acid (HDACi)	Viral RNA < 50cp/mL for at least 6 months; healthy CD4 T-cells >300/μL	12	Single arm phase 1 trial	1000 mg qd (Depakote ER)	16 weeks	No sustained depletion of resting CD4+ T-cell infection observed
Routy et al. 2012	Valproic Acid (HDACi)	Viral RNA < 50cp/mL for at least 1 year; healthy CD4 T-cells >200/μL	56	Multicenter randomized cross-over trial	Up to 500 mg bid (as per tolerance)	16 weeks (× 2)	No reduction in CD4 T-cells with replication-competent HIV
Elliot et al. 2014	Vorinostat (HDACi)	Not reported Baseline CD4 T-cell count range from 371 to 1136	20	Proof-of-concept single arm	400 mg bid for 14 days	12 weeks	Cell-associated unspliced RNA Increased by 7.4 fold at 14 days
Archin et al. 2013	Vorinostat (HDACi)	Viral RNA < 50cp/mL for at least 6 months; healthy CD4 T-cells >300/μL	11	Proof-of-concept single arm	200 mg initially 400 mg after 2–3 and 4–5 weeks	5 weeks	RNA expr. in resting CD4+ T-cells increased 4.8 fold (1.5–10)
Rasmussen et al. 2014	Panobinostat (HDACi)	Viral RNA < 50cp/mL for at least 2 years; healthy CD4 T-cells >500/μL	15	Phase 1/2	20 mg three times/week for 8 weeks	32 weeks	Cell-associated RNA Increased during treatment by 3.5 fold
Soegaard 2015	Romidepsin	Viral RNA < 50cp/mL for at least 2 years; healthy CD4 T-cells >500/μL	6	Proof-of-concept phase II	One 4 h 50 mg infusion per week for 3 weeks	70 days after last infusion	Plasma RNA increased to detectable levels in 5/6 patients
Katlama 2016	IL-7 agonist Raltegravir Maraviroc	Viral RNA < 200cp/mL last 3 years, <50cp/mL last 6 months, CD4 T-cells >350/μL	29	Randomized trial	8 weeks of RAL + MAR intensification, then 3 x weekly 30mcg/kg of IL-7	28 weeks	Data safety monitoring board stopped trial at 28 weeks due to concerns of >1500 CD4+ T-cell counts
Levy Y 2012	IL-7 recombinant	Viral RNA < 50cp/mL for at least 6 months; healthy CD4 T-cells: 100–400/μL	32	Randomized trial	weekly 10, 20, or 30mcg/kg for 3 weeks	52 weeks	IL-7 well tolerated up to 20mcg/kg. Brisk increase in CD4 count.
Sereti 2009	IL-7 recombinant	Viral RNA < 50cp/mL (grp 1) Viral RNA < 50,000cp/mL (grp 2) for at least 12 months; healthy CD4 T-cells > 100/μL	25	Randomized double blind	Single dose of 3, 10, 30, 60 or 100μg/kg	8 weeks	30μg/kg max tolerable dose. Significant increase in transient HIV-RNA in 6 patients. Increase in central memory phenotype T-cells
Vibholm 2017	TLR-9 agonist (MGN1703)	Viral RNA < 50cp/mL for at least 12 months; healthy CD4 T-cells >350/μL	15	Phase 1/2a Single arm	60 mg MGN1703 subcutaneously twice weekly for 4 weeks	4 weeks (80 days follow-up)	Pronounced activation of plasmacytoid dendritic cells. Significant increase in proportions of activated cytotoxic NK cells and CD8+ T cells
Epigenetic modulation agents							
Elliott et al. 2015	Disulfram	Viral RNA < 50cp/mL for at least 3 years; healthy CD4 T-cells >350/μL	30	Non-randomized prospective dose-escalation	Three days of 500 mg, 1000 mg, or 2000 mg	30 days	Approximately 2-fold increase in cell-associated RNA
Spivak et al. 2014	Disulfram	Viral RNA < 50cp/mL for min 1 year; healthy CD4 T-cells >500/mcg for min 24 weeks	16	Pilot single arm	500 mg/day for 14 days	84 days	Well-tolerated, but latent reservoir did not change in size
Gutiérrez et al. 2016	Bryostatin-1	Viral RNA < 50cp/mL for at least 2 years; healthy CD4 T-cells >350/μL	12	Double-blind randomized phase I trial	Placebo 10mcg/mm^2^ 20mcg/mm^2^	672 days	No detectable difference in cell-associated unspliced RNA

#### Agents for targeting cellular transcription factors

With respect to ‘kick’ agents targeting cellular transcription factor, studies have included Protein Kinase C agonists (e.g. prostratin, bryostatins), which activate the canonical NF-αB pathway ultimately resulting in initiation of viral transcription [15, 23, 24]. However, these agents are generally toxic and clinical studies involving human participants have shown very little effect at tolerable doses [25]. Hexamethylene bisacetamide (HMBA) and Disulfram have been identified as affecting latency reversal by stimulating the release of positive transcription elongation factor b (P-TEFb) (from Hexim-1 and 7SK snRNA) via the Akt pathway [26–29]. P-TEFb catalyses phosphorylation of a number of transcriptional regulators at the HIV promotor site which support transcriptional initiation and elongation. Experiments with these pharmaceuticals in vitro showed promise, but two clinical trials of Disulfram in humans have yielded only a modest activation of the latent reservoir with indeterminate evidence of a reduction in the T-cell latent reservoir [30, 31]. To our knowledge, no future trials on disulfram are planned [32]. Interleukin (IL)-7 is required to stimulate homeostatic proliferation of resting memory T-cells, and it has therefore been hypothesized that IL-7 could potentially act as a latency reversing agent for resting infected CD4+ T-cells. To date, results in humans have been conflicting. One randomized placebo controlled dose-response trial adding recombinant human IL-7 to current ART in 32 patients with with low CD4+ T-cell counts (101–400 cells/mcg) showed an increase in thymic output, improved T-cell receptor repertoire, and increased cell cycling and bcl-2 expression [33]. By contrast, another randomized clinical trial combining IL-7 with raltegravir and maraviroc in 29 patients with CD4+ T-cell counts >350cells/mcg did not show any change in HIV DNA in peripheral blood mononuclear cells [34]. This negative finding is not surprising since other studies have shown that IL-7 contributes to viral persistence, leading to proliferation of infected cells and is not sufficient in reversing latency in quiescent T cells [35, 36]. Currently, one future IL-7 clinical trial is planned in which IL-7 will be combined with LIPO 5 DC, an experimental dendritic cell based therapeutic vaccine. Another class of agents, toll-like receptor (TLR) agonists, have been shown induce HIV expression and HIV specific immunity in patients receiving ART [32]. The latency reversing properties associated with the TLRs comprise activation via the NF-κB, NFAT, or AP-1 pathways [37, 38]. In addition, stimulation of TLR-7 have been shown effective as an adjuvant to therapeutic vaccination in SIV-infected rhesus monkeys [39]. Currently, one single arm (phase I) clinical trial has investigated TLR-agonists, namely the novel MGN1703 TLR-9 agonist in 15 virologically suppressed HIV infected individuals [40]. In this trial, CD8+ T-cells and natural killer (NK) cells significantly increased during treatment, thus suggesting enhanced immune response to the virus. In 6 of the 16 participants viral RNA copies also increased from <20 to >1500 copies/mL, potentially suggesting re-activation of the latent reservoir. Two more clinical trials are being planned to investigate the latency reversing effects of TLR agonists [32].

#### Epigenetic modulation agents

In the case of epigenetic modulation, the histone deacetylase (HDAC) inhibitors are the most studied pharmaceutical agents to reactivate the latent reservoir. These studies have received comprehensive review by others [41, 42]. Briefly, in the latent state, HDAC enzymes accumulate around the HIV promotor site favouring deacetylation of the associated nucleosomes and hence restricting HIV replication. Researchers have demonstrated a number of different HDAC inhibitors (e.g. valproic acid, vorinostat, panobinostat, Romidepsin, suberoyl bis-hydroxamic acid) stimulate transcription, thus activating the latent cells both in vitro and in vivo. In clinical trials, however, treatment with these agents resulted in no significant reduction of the latent viral reservoir of memory T-cells although there was evidence that infected cells were being activated (see Table 1). Despite disappointing results in these early clinical trials, a total of nine trials examining these agents, either alone or in combination with immune boosters, are planned or ongoing [32].

Another class of compounds that have been examined as latency reversing agents include Histone methyl transferase inhibitors (chaetocin and BIX-01294) [43, 44]. These agents affect histone methylation and/or demethylation stimulating or repressing transcription in a manner akin to the deactylase inhibitors. Also, the DNA cytosine demethylation agent 5-aza-2-deoxycytidine (Aza-CdR) can reverse latency by demethylating CpG islands Regions of DNA usually found near gene promotor sites with high density of cytosine-phosphate-guanine (CpG) clusters in the HIV transcription initiation site [45]. All these agents have been shown to reverse latency in vitro and/or ex vivo, but have not been tested in vivo as far as we are aware [43–45].

### The missing ‘kill’ in ‘kick and kill’

While there are a number of factors that might explain the failure of the latency reversal agents to meaningfully impact the T-cell reservoir in initial studies, it is important to recognize that the entire kick and kill paradigm is based on the premise that latently infected cells will either expire due to virus induced lysis/apoptosis or be destroyed by cytotoxic T-cell (CTL) response soon after activation. Recent evidence, however, suggests that this may not be the case [35, 46, 47]. For example, in one study of latency reversal in CD4^+^ T-cells obtained from HIV-infected donors, Shan and colleagues found that reactivation of latent infected cells by the HDAC inhibitor vorinostat did not affect the reservoir [47]. Furthermore, the addition of CD8^+^ T cells from patients on suppressive ART did not induce cell death. Another study suggested that the HDAC inhibitors in general may suppress the CTL response. In particular, it was observed that vorinostat, panobinostat and romidepsin impaired the ability of HIV-specific CTL to eliminate infected CD4^+^ T-cells ex vivo [46]. Other data suggest that the vigorous CTL response observed during acute infection may be lost during chronic infection, and thus, that CTL response might be impaired independent of administration of HDAC inhibitors [48]. Collectively, these observations suggest that ‘Kicking’ alone is likely insufficient to eliminate the latent T-cell reservoir.

Another limitation of current kick and kill clinical studies is the almost ubiquitous focus on T-cell reservoirs. By now, it has been well-document that several other secondary reservoirs may be a key contributor to the persistence of the HIV virus. To this end, we have already discussed the likely significant role that monocytes and macrophages play in the persistence of the HIV virus. As these cells are known to be relatively resistant to the cytopathic effect of the HIV infection, stimulating them may only serve to increase the production of virions and/or associated viral proteins. Another latent reservoir that is believed to sustain viral transmission during ART are the dendritic cells. Dendritic cells can shelter viral particles within endosomal compartments or carry multiple particles attached out outer membrane synapses. Dendritic cells can live for several years, during which they can slowly gather increasing amounts of viral particles [49]. To our knowledge, interaction of latency reversing agents and dendritic cells has not been explored. However, since dendritic cells generally do not integrate or replicate viral RNA, latency reversing agents are not expected to affect dendritic cells. Although controversy still exists on the importance of dendritic cells as a component of the latent reservoir, with our current understanding, one could argue a feasible and efficient kill strategy targeting dendritic cells might be essential in attaining a cure.

### Kill strategies

With recent results suggesting the simple reversal of latency (kick) strategy to be insufficient for purging the latent reservoir, it is time to look more closely at approaches that focus on killing reactivated cells. Such agents might have the potential to act directly on latent cells removing the need for the latency reversing drug. Here, we look at some approaches that focus on ‘the Kill.’

#### Broadly neutralizing monoclonal antibodies

**Fig. 1:**
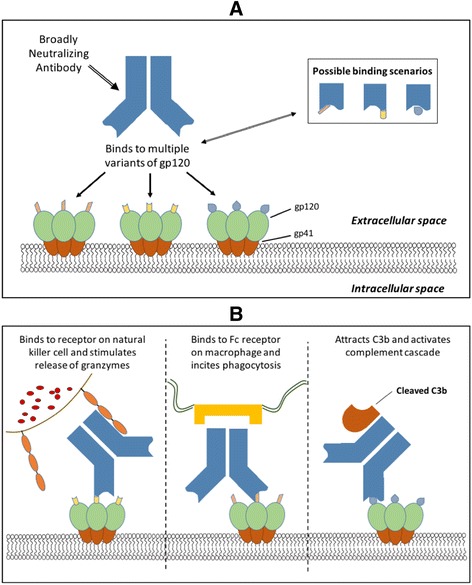
Representation of ability broadly neutralizing antibody to (**a**) bind to multiple variants of gp120; (**b**) induce killing of the HIV infected cell by attraction of natural killer cells (*left*), macrophages (*middle*) and complement (*right*)

Application of broadly neutralizing monoclonal antibodies (mAb) for prevention, post exposure prophylaxis and the treatment of HIV have has been increasingly pursued since the advent of single cell based cloning methods has made isolation of mAb against the virus mainstream [50–53]. These proteins target the virus envelope proteins composed of multiple HIV gp120 surface proteins coupled to gp41 transmembrane proteins. For treatment purposes, there are numerous mAb being studied; some exhibiting significant breadth and potency against hundreds of HIV variants [54, 55]. Fig. 1 illustrate the general mechanism of action associated with broadly neutralizing monoclonal antibodies for the treatment of HIV. To date, two of these have advanced to clinical trials in human participants. In the first case, administration of a single dose (30 mg/kg) of 3BNC117 (which targets the CD4^+^ binding site of the viral spike) to viremic subjects (*N* = 17, 2 on ART) resulted in a 0.8–2.5 log10 decrease in viremia which persisted for 28 days [56]. Similarly, six of eight subjects treated with the CD4^+^ blocking monoclonal VRC01 (40 mg/kg, IV) experienced a 1.1 to 1.8 log_10_ reduction in plasma viremia. In this latter case, the two non-responders were shown to be infected with resistant variants prior to treatment. This observation highlights the importance that resistance avoidance will be critical if these therapies are to be successful. In both trials, resistant viral variants emerged in at least some patients after clearance of the antibodies. Further, studies in animal models indicate changes in just one to three target protein residues is enough to allow for viral escape [54]. Thus, as it is with ART, combinations of mAbs targeting different epitopes will likely be required for clinical application and early studies both in vitro and in animal models suggest that this could be a viable approach [57, 58].

Until recently, the primary focus of studies around the therapeutic use of mAb for HIV has been on active infection and the ability of mAbs to block viral infiltration into cells. However, these agents also have potential to orchestrate the destruction of the latent reservoir via antibody-dependent cell-mediated cytotoxicity (ADCC) [59]. ADCC is mediated by the antibody Fc chain which can recruit natural killer (NK) cells, macrophages, polymorphonuclear phagocytes or complement. Recent studies suggest that some of the broadly neutralizing mAb appear to direct ADCC in latent HIV-infected T-cells [60]. This has been demonstrated in vitro with multiple broadly neutralizing mAbs (including 3BNC117 and VRC01) where research demonstrated varying degrees of ADCC in laboratory strains (CD4^+^ lymphoid cells (MT4) infected with the prototypic R5-tropic NLAD8 or X4-tropic NL4.3) and primary HIV isolates. Of the mAbs tested in laboratory strains, 5 strongly induced ADCC (NIH45–46, 3BNC117, 10–1074, PGT121, 10E8), two less so (PG16, VRC01) with the balance being inactive. With respect to primary HIV isolates obtained from infected patients, the effect was somewhat attenuated requiring a combination of 5 mAbs (NIH45–46, 3BNC117, 10–1074, PG16 and 10E8, each at 1.5 mg ml) to induce cell death in some but not all strains. The reduced activity was associated with a reduced number of available binding sites in the infected cells. Interestingly, the same combination of five mAbs induced cell death ex vivo in reactivated (by phytohemagglutinin PHA) latent CD4^+^ isolates obtained from 5 out of 6 ART suppressed HIV infected subjects.

Direct in vivo studies of mAb induced ADCC are limited to animal models [60, 61]. In a study of HIV infected (HIV-1_YU2_) humanized mice, researchers found that animals treated with mAb mixture (3BNC117, 10–1074, and PG16) were slower to rebound upon withdrawal of treatment relative to ART treated mice (74–107 days vs 28–84 days) [60]. This finding was interpreted as an indication of a reduced latent reservoir mediated via Fc mediated ADCC [54]. This was supported using the same combination of mAb, but with the Fc effector function removed. Here, nine of 15 of the mice on the knock out regimen rebounded within 44 days after cessation of therapy relative to 1 of 21 mice receiving the unmutated mAb (*p* = 0.0004). Further, rebound viremia was 50-fold higher in the mice on the knock out mAb (*p* = 0001).

In humans, evidence of ADCC is indirect. Results from the RV144 trial vaccine trial showed a 31% reduction in HIV acquisition (*P* = 0.04), however, the antibodies induced by the vaccine did not suppress primary HIV isolates and it was therefore theorized that the observed effect might be associated with ADCC [62]. Modelling studies of passively administered 3BNC117 also suggest that the observed results cannot be attributed to clearance of free virus alone and that Fc mediated ADCC likely a contributing factor [61]. Taken together all these results suggest that addition of mAbs to the kick and kill strategies deserve investigation.

#### Integrin receptor targeted antibody therapy

In a recent report, an antibody therapy targeting CD4+ T-cell proteins appeared to confer impressive viral control in primates recently infected with Simian immunodeficiency virus (SIV) [63]. In this study, ART-treated SIV-infected rhesus macaques received eight infusions of a primatized monoclonal antibody against the α4β7 integrin both during ART, and for a period after discontinuing ART. After cessation of ART, all eight test animals achieved viral control (low to undetectable levels) after a period of modest viral rebound (note: 2 of 8 never exhibited rebound). Virologic control was sustained for over 45 weeks after discontinuing ART. This is in contrast to the seven macaques in the control arm (which received nonspecific rhesus immunoglobulin G instead of α4β7 mAb) where all the animals rebounded to high viral loads (6 logs) within 2 weeks of stopping ART. Furthermore, CD4+ T-cell counts recovered to ‘healthy’ levels in the α4β7 mAb treated animals soon after the first administration of the antibody and remained stable for over 25 weeks off all therapy. There was no recovery in the controls.

The mechanism for the observed effect of the anti-α4β7 integrin in this study is not entirely clear. The α4β7 integrin is a CD4 cell surface protein that is instrumental in the trafficking of these cells to the gastrointestinal tissue. During acute infection, a great deal of damage occurs in the gastrointestinal tissue including a precipitous drop in CD4+ T-cells, damage to the intestinal epithelium and the rapid establishment of the viral reservoir. It was believed that limiting access of CD4+ T-cells to the gut with the anti-α4β7 might mitigate this damage. Whether that explains the results can not be determined from this study as CD4^+^ counts in the small intestine began to recover during the period of administration of the anti-α4β7. Another potential mechanism may be the property that anti-α4β7 monoclonal antibodies allow for the production of anti-v2 antibodies, which have been shown to mediate antibody-dependent cellular phagocytosis (ADCP), and thus contribute to the suppression of the proliferation of infected cells [64]. Collectively, the observations of this study may be important to cure research although it is not clear if they would extend to later stages of disease.

#### BiTEs and DARTs

Bispecific T-cell Engagers (BiTEs) and Dual-Affinity Re-targeting Molecules (DARTS) are variations of bispecific antibodies, which are engineered with the binding regions of two different antibodies such that they bind two different antigens. Both BITEs and DARTs belong to a class of these compounds which exclude the antibody Fc region. Progress in the use of these agents for the treatment of cancer has recently spurred research in their application to the treatment of HIV-1.

**Fig. 2:**
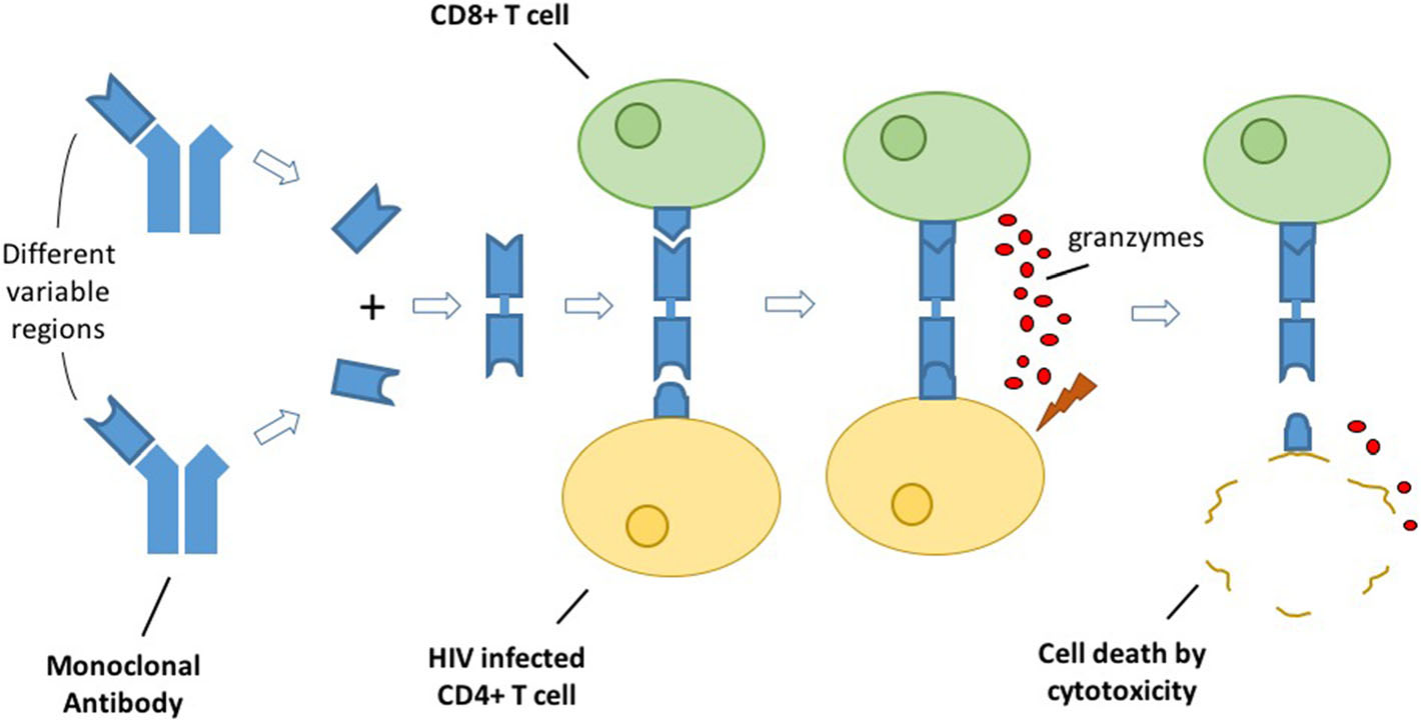
Assembly of BiTEs from two different variable regions of monoclonal antibodies and their mechanism of action. The BiTE first attaches to a CD8+ T cell before assisting the CD8+ T cell in binding to an HIV infected CD4+ T cell. Upon binding the CD4+ T cell the CD8+ T cell will release granzymes and induce death of the HIV infected CD4+ T cell

BiTEs are comprised of two antibody single chain variable fragments linked together by a short flexible peptide [65]. One fragment is targeted towards CD3 protein of the T-cell receptor complex which signals T-cell effector functions. In a recent study of a BiTE incorporating the light chain of the broadly neutralizing antibody VRC07, researchers found that the resulting VRC07-CD3 BiTE induced CTL elimination of latently infected cells isolated from peripheral blood mononuclear cells (PBMCs) of infected donors ex vivo [66]. Fig. 2 illustrates, in brief, the assembly process and the general mechanism of action associated with BiTEs for the treatment of HIV.

In subtle contrast to BiTEs, DARTs are constructed from the variable heavy domain of one antibody linked to the light variable domain of another [55]. DART proteins have shown to mediate CTL clearance of latently infected CD4^+^ T-cells both in vitro and ex vivo [67, 68]. Thus, proof-of-concept of these agents as potential kill agents seems to have been met. It will be interesting if these results can be recapitulated in vivo.

#### Chimeric antigen receptors (CARs)

**Fig. 3:**
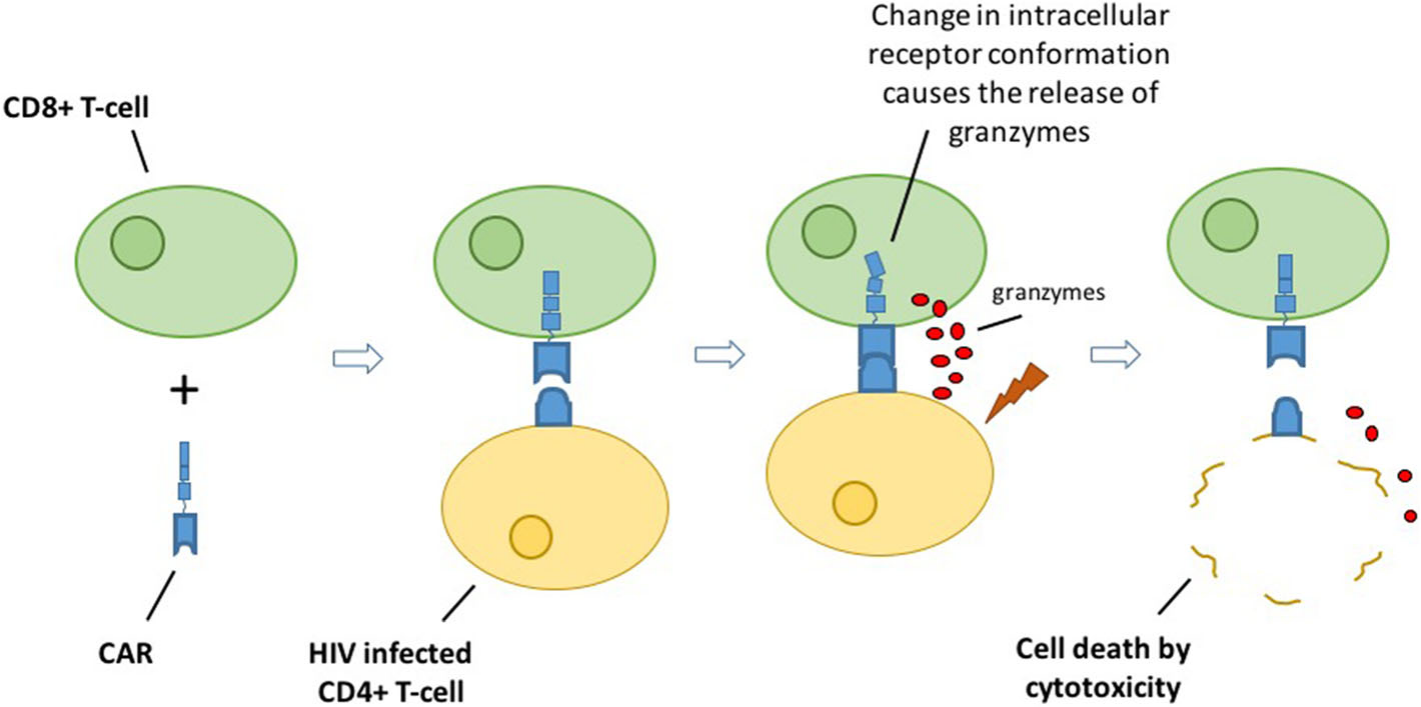
Assembly of a CAR engineered CD8+ T-cell its mechanism of action. The CAR engineered CD8+ T cell binds to an HIV infected CD4+ T cell. Upon binding the CD4+ T cell the CAR engineered CD8+ T cell will release granzymes and induce death of the HIV infected CD4+ T cell

Another class of kill agents with renewed excitement are HIV specific T-cells engineered with chimeric antigen receptors (CARs) [69]. CAR receptors are comprised of a target specific surface protein coupled to an intracellular signalling domain to activate the cytotoxic response. The first CAR was based on a soluble CD4^+^ receptor (intended to bind to infected cells expressing HIV gp120) coupled to an intracellular CD3 ζ signalling protein. In vitro, these designer cells were as effective at killing infected cells and CTL clones isolated from infected patients [70]. Unfortunately, when tested on HIV infected subjects, they had no effect on clinical outcomes (although they were well tolerated and persisted for years). Because of this, CAR research was abandoned. However, recent progress in treatment of cancer with CARs coupled with the discovery of broadly neutralizing antibodies (which serve as CAR receptor models) has renewed interest in their application to HIV renewed [71]. In vitro*,* these antibody-modelled CARs show promise, but it remains to be seen if they will be effective in vivo. Fig. 3 illustrates the assembly of a CAR engineered T cell and the general mechanism of action associated with CARs for the treatment of HIV.

#### Second mitochondria-derived activator of Caspases

Second Mitochondria-derived Activator of Caspases (SMAC) mimetics are small molecule drugs that show potential to induce cell death in reactivated latent cells. Caspases are proteolytic enzymes involved in apoptosis and IAPs (Inhibitors of Apoptosis Protein) are regulatory proteins that inhibit caspase activity by either binding directly to the enzyme (e.g. XIAP) or by blocking signals that lead to their activation (e.g. IAP2, IAP3). SMAC is an endogenous protein that suppresses the activity of the IAPs thus promoting cell death. Several small molecule drugs fashioned after the key binding domain of SMAC have been developed to combat apoptosis resistant cancer cells with some already entering clinical trials [72]. While studies are still in the early stages, researchers are currently exploring these agents to eliminate the HIV latent reservoir. In a recent report, in vitro treatment of HIV infected central memory T-cells with 3 SMAC mimetics (birinapant, GDC-0152 and emblin) targeting XIAP activity successfully induced a significant dose-dependent increase in apoptosis [73].

Another study of SMAC mimetics demonstrated latency reversing abilities by looking at SMAC inhibitors of IAP1 and IAP2, two proteins that are known to inhibit NF-κB inducing Kinase (NIK) by ubiquitination. In the absence of IAP1 and IAP2, NIK will accumulate, initiating a series of reactions that result in the activation of NF-κB which can translocate to the nucleus and initiate HIV transcription. Researchers tested four SMAC mimetics (SBI-0637142, LCL161, GDC-0152, TL32711) specifically targeting IAP1 and/or IAP2 and all exhibited latency reversing capacity in a Jurkat latency model [74]. SBI-0637142 and LCL161 were also tested ex vivo in CD4+ T-cells collected from HIV patients on suppressive ART, and while neither seemed to initiate activation of these cells on their own, there was a potent synergistic effect when they were used in combination with the HDAC inhibitor panobinostat. Taken together, these results suggest an exciting potential for SMAC mimetics in kick and kill strategies.

#### Immune checkpoint antibodies

The role of programmed cell death receptor 1 (PD-1) and PD1 ligand (PD-L1) expression in HIV patients have been investigated in several studies [75]. Elevated expression of PD1 has been demonstrated in both HIV specific CD8+ T cells and CD4+ T-cells. In particular, the interaction of PD-1 and PD-L1 are suspected to be a major contributor to the persistence of infected CD4+ T-cells [76]. High PD1 expression has been shown correlated with the exhaustion of CD8+ T-cells [77]. Antibodies for PD-L1 ligand have been developed for several late stage cancers and these have shown effective in inducing natural apoptosis and increasing overall survival. Case studies of cancer patients with HIV receiving PD-L1 antibodies have also reported promising results such as substantial lowering of viral loads [78]. Given the promise of these early results, clinical trials have been initiated. In one completed 8-person phase trial (6 patients receiving one infusion of nivolimumab 0.3 mg/kg, 2 patients receiving normal saline as placebo), two of the nivolimumab treated patients showed evidence of reversal of CD8+ T-cell exhaustion 4 weeks after the infusion [79]. Two larger trials are currently ongoing [32].

#### Therapeutic vaccination

While most vaccine research has focussed on prevention, numerous clinical studies of therapeutic vaccines in HIV infected subjects have been conducted [80, 81]. Some of these have shown modest drops in viral load (0.5 to 1 log drop) using various approaches to boost the immune system, and thus, can arguably be considered ‘kick’ strategies. For example, in the single arm REDUC study, subjects received multiple doses of Vacc-4× (a synthetic gag peptide) with recombinant humanized granulocyte macrophage colony-stimulating factor (rhu-GM-CSF) followed by administration of the HDAC inhibitor romidepsin [82]. The rationale for this study was based on previous data that suggested the Vacc-4×/rhu-GM-CSF vaccine induced killing of infected cells. While the results of the treatment showed a significant (*p* = 0.01) 40% reduction in the proviral DNA, it did not have any effect in time to rebound after treatment interruption. Researchers concluded the treatment required fine tuning. Another vaccine trial, the BCN01 trial, suggested that a combination of the ChAd.HIVconsv and the MVA.HIVconsv prime boost vaccines are efficacious in redirecting CD8+ T-cell response towards regions where HIV-1 is highly conserved [83]. Further, a recent extension study of BCN01, the BCN02 proof-of-concept study, in which another boost with MVA.HIVconsv was followed by three weekly 5 mg/m^2^ doses of romdepsin and a second MVA.HIVconsv boost, demonstrated highly promising results as an therapeutic strategy that encompasses both a kick and a kill component [84]. Of the 15 patients enrolled in this proof-of-concept study, the most recently presented data (CROI, February 2017) revealed that four patients have remained off ART for 7, 12, 14, and 22, weeks respectively. Longer term follow-up results cast further light on the efficacy of this therapeutic combination.

### Conclusions

For patients, clinicians and healthcare funders alike, developing a sterilizing HIV cure which completely clears the virus would be the ultimate goal. However, a ‘functional cure’ that would allow the body to control the virus in the absence of other treatments (i.e. ART) for a considerable duration of time is generally considered more realistic. A functional cure would provide much needed relief to patients on rigorous daily antiretroviral regimens. Further, it would likely have a greater impact than conventional ART in settings where adherence or frequent access to medication presents a challenge. ‘Kick and kill’ cure approaches have taken the lead in cure research, but to date results have been disappointing. This may stem from the fact that previous approaches have not taken full advantage of available kill approaches, and thus missed out on the opportunity to kill a sufficient quantity of re-activated cells or even the resting cells themselves. Here we have reviewed five kill approaches that show potential to reduce or eliminate the latent reservoir. These might be used alone, in combination with each other and/or in combination with latency reversing agents. Given the complexity of HIV infection and the multiplicity of compartments (cellular and tissue) involved, it seems highly unlikely that there will be a single ‘magic cure bullet’. Instead, the cure is almost certainly going to require a multipronged approach involving new drug combinations. While an understanding of the efficacy and safety of each potential component is crucial, we believe it is paramount that future research includes an additional focus on finding the best combination of therapies to clear infected cells.

## Abbreviations

ADCC: Antibody-dependent cell-mediated cytotoxicity
AIDS: Acquired Immunodeficiency Syndrome
ART: Antiretroviral therapy
BITE: Bispecific T-cell Engagers
CAR: Chimeric antigen receptors
CTL: Cytotoxic T-cell
DARTS: Dual-Affinity Re-targeting Molecules
EC_50_: Half maximal effective concentration
HDAC: Histone deacetylase
HIV: Human immunodeficiency virus
II: Integrase inhibitors
IL: Interleukin
mAb: Monoclonal Antibody
NNRTI: Non-nucleoside reverse transcriptase inhibitors
NRTI: Nucleoside reverse transcriptase inhibitors
PD1: Programmed cell death receptor 1
PD-L1: PD1 ligand
PI: Protease inhibitors
P-TEFb: Positive transcription elongation factor b
SIV: Simian immunodeficiency virus
SMAC: Second Mitochondria-derived Activator of Caspases
TLR: Toll-like receptor
